# Supplementary material for: Preferred temperature and thermal breadth of birds wintering in peninsular Spain: the limited effect of temperature on species distribution
Source: PeerJ. 2016 Jul 19;4:e2156. doi: 10.7717/peerj.2156 (PMC4957994; doi:10.7717/peerj.2156)
Supplement: Text S1 [file peerj-04-2156-s001.docx]

**Supplemental Text S1**. **Description of the Spanish Bird Atlas of Winter Birds.**

Hundreds of experienced birdwatchers were asked to sample UTM cells following a standardized methodology during three consecutive winters (2007-2010, from mid-November to mid-February), in order to map the distribution and relative abundance of each species throughout peninsular Spain. The sample unit was the 10x10 km UTM cell, so that participants had to accumulate a minimum survey of 15 h per cell, divided in 60 line transects of 15-min, and covering all the habitats present in proportion to their extent in the 100 km^2^ (previously calculated by means of GIS tools). This relative frequency is highly correlated with standardized bird counts (i.e., birds detected per km in each UTM cell), thus accurately informing about spatial variation in bird abundance (Palomino et al. 2007; SEO/BirdLife, 2012). The final sample size was 1,689 UTM cells, c. 80% out of 2,121 UTM cells covered in the Atlas, after discarding those with too little sampling effort (less than 60 line transects per 100 Km^2^ in the three winters), and low land surface in peninsular Spain (<50 Km^2^).

Palomino, D., Carrascal, L.M., Del Moral, J.C. (2007) Atlas de las aves invernantes en España. Boletín N.º 1. SEO/BirdLife. Madrid.
