# Supplementary material for: Preferred temperature and thermal breadth of birds wintering in peninsular Spain: the limited effect of temperature on species distribution
Source: PeerJ. 2016 Jul 19;4:e2156. doi: 10.7717/peerj.2156 (PMC4957994; doi:10.7717/peerj.2156)
Supplement: Table S1 [file peerj-04-2156-s003.docx]

**Supplemental Table S3.** **Parameters defining the relationship between relative abundance and minimum winter temperature utilized in the study and obtained for 103 terrestrial bird species wintering in the Iberian Peninsula, corresponding to winter censuses on 1689 10x10 km^2^ UTM (years 2008-2011).** **#UTM**: number of UTM cells where the species were present. **ΔAIC:** increase in Akaike value AIC of 95% quantile regression models, comparing the null model (only the intercept *a*) with the model including the linear (*b*) and quadratic (*c*) terms of average winter minimum temperature (using the standardized values of these terms; i.e., at mean = 0 and sd = 1). **pR^2^50, pR^2^75, pR^2^90 and pR^2^95**: pseudo-R^2^ (in percentage) of the quadratic quantile regression models between the relative abundance of each species and winter minimum temperature, using the percentiles 50%, 75%, 90% and 95%, respectively. ***a*:** intercept of the quantile regression, a measure indicating the relative abundance of each species at the percentile 95% (the maximum attainable figure is 60). ***b*, *c*:** standardized linear and quadratic regression coefficients obtained for the 95% quantiles. **T_PREF_:** environmental preferred temperature (minimum winter temperature at which the maximum relative abundance of the species is predicted by 95% quantile regression models). **T_BREADH_:** thermal breadth (area under the curve from -2 to 10 ºC of 95%-quantile regression equations with the maximum abundance of each species relativized to 1). **T_MEAN_:** mean temperature (mean of average winter minimum temperature in those UTM cells where the species were present, weighed by their relative abundances). **sd:** weighed standard deviation of T_MEAN_. **t test:** t statistic comparing the observed figure of T_MEAN_ with the average winter minimum temperature in all studied UTM cells (2.55 ºC). **p:** significance of t tests (in bold type, those significance values remaining significant after sequential Bonferroni correction). *a*, *b*, *c* and T_PREF_ are only presented for those significant models with a reduction in AIC figures (ΔAIC) lower than -13.82 units (i.e., the polynomial 95% quantile model was 1,000 times better in explaining the variation in relative abundance of the species than the null 95% quantile model: exp [-0.5*ΔAIC]).

| Species | #UTM | ΔAIC | pR^2^50 | pR^2^75 | pR^2^90 | pR^2^95 | *a* | *b* | *c* | T_PREF_ | T_BREADH_ | T_MEAN_ | sd | t test | p |
| --- | --- | --- | --- | --- | --- | --- | --- | --- | --- | --- | --- | --- | --- | --- | --- |
| *Accipiter gentilis* | 261 | 4.0 | 0.0 | 0.0 | 0.0 | 0.0 |  |  |  |  | 1.00 | 2.0 | 2.32 | -3.89 | **0.000** |
| *Accipiter nisus* | 804 | 4.0 | 0.0 | 0.0 | 0.0 | 0.0 |  |  |  |  | 1.00 | 2.5 | 2.38 | -1.13 | 0.258 |
| *Aegithalos caudatus* | 1352 | -112.5 | 0.9 | 1.5 | 2.8 | 3.4 | 17.8 | 4.39 | -6.02 | 2.4 | 0.74 | 2.3 | 2.25 | -3.60 | **0.000** |
| *Aegypius monachus* | 185 | -255.7 | 0.0 | 0.0 | 3.5 | 7.4 | 2.2 | 1.89 | -1.62 | 3.9 | 0.62 | 3.2 | 1.58 | 5.24 | **0.000** |
| *Alauda arvensis* | 1121 | -41.2 | 0.9 | 1.8 | 1.4 | 1.3 | 31.7 | -6.67 | 3.33 | -2.0 | 0.73 | 2.1 | 2.34 | -6.19 | **0.000** |
| *Alcedo atthis* | 218 | -388.3 | 0.0 | 0.0 | 9.4 | 11.0 | 1.4 | 0.65 | 0.42 | 10.0 | 0.39 | 4.5 | 2.15 | 13.29 | **0.000** |
| *Alectoris rufa* | 1298 | -322.3 | 0.5 | 3.7 | 7.7 | 9.2 | 23.4 | 14.33 | -9.42 | 5.1 | 0.76 | 3.1 | 2.43 | 8.09 | **0.000** |
| *Anthus pratensis* | 1426 | -486.5 | 10.4 | 13.9 | 12.9 | 13.5 | 30.0 | 11.92 | -4.30 | 9.3 | 0.76 | 3.6 | 2.42 | 15.85 | **0.000** |
| *Anthus spinoletta* | 199 | 4.0 | 0.0 | 0.0 | 0.0 | 0.0 |  |  |  |  | 1.00 | 2.5 | 2.44 | -0.08 | 0.933 |
| *Aquila chrysaetos* | 384 | 3.6 | 0.0 | 3.1 | 0.0 | 0.0 | 1.9 | 0.10 | -0.40 | 0.8 | 0.74 | 1.9 | 2.11 | -5.70 | **0.000** |
| *Burhinus oedicnemus* | 177 | -596.4 | 0.0 | 0.0 | 16.5 | 16.3 | 1.3 | 0.60 | 0.39 | 10.0 | 0.39 | 5.0 | 2.19 | 14.59 | **0.000** |
| *Buteo buteo* | 1372 | -34.8 | 2.0 | 2.1 | 1.6 | 1.1 | 13.3 | 1.93 | -3.05 | 2.1 | 0.80 | 2.4 | 2.24 | -3.08 | 0.002 |
| *Carduelis cannabina* | 1373 | -2.6 | 0.0 | 0.2 | 0.1 | 0.2 |  |  |  |  | 0.93 | 2.5 | 2.49 | -0.70 | 0.486 |
| *Carduelis carduelis* | 1591 | -383.6 | 6.5 | 8.3 | 11.6 | 10.8 | 28.6 | 4.50 | 1.39 | 10.0 | 0.66 | 3.3 | 2.61 | 10.98 | **0.000** |
| *Carduelis chloris* | 1362 | -626.1 | 9.0 | 15.6 | 20.0 | 17.0 | 16.4 | 1.62 | 4.36 | 10.0 | 0.48 | 3.7 | 2.75 | 16.01 | **0.000** |
| *Carduelis spinus* | 728 | -38.4 | 0.0 | 0.3 | 0.9 | 1.3 | 7.6 | 0.65 | 0.37 | 10.0 | 0.72 | 2.9 | 2.55 | 3.80 | **0.000** |
| *Certhia brachydactyla* | 1094 | -5.4 | 0.3 | 0.0 | 0.4 | 0.3 |  |  |  |  | 0.72 | 2.5 | 2.51 | -0.68 | 0.497 |
| *Cettia cetti* | 704 | -104.6 | 0.5 | 3.1 | 3.0 | 3.2 | 5.9 | 1.49 | 0.24 | 10.0 | 0.60 | 3.4 | 2.67 | 8.77 | **0.000** |
| *Cinclus cinclus* | 176 | -296.0 | 0.0 | 0.0 | 8.5 | 8.5 | 1.3 | -0.97 | 0.30 | -2.0 | 0.35 | 1.1 | 2.05 | -9.38 | **0.000** |
| *Circus aeruginosus* | 286 | -56.4 | 0.0 | 0.0 | 1.0 | 1.8 | 3.6 | 2.24 | -1.53 | 4.9 | 0.75 | 3.3 | 2.61 | 4.82 | **0.000** |
| *Circus cyaneus* | 568 | -103.8 | 0.0 | 1.6 | 3.3 | 3.1 | 3.3 | -1.14 | 0.41 | -2.0 | 0.58 | 1.7 | 2.16 | -9.70 | **0.000** |
| *Cisticola juncidis* | 482 | -1296.6 | 1.0 | 16.5 | 26.9 | 32.0 | 5.9 | 0.38 | 4.58 | 10.0 | 0.33 | 5.2 | 2.51 | 22.92 | **0.000** |
| *Coccothraustes coccothraustes* | 359 | -7.6 | 0.0 | 0.8 | 2.1 | 0.3 | 3.1 | -0.71 | 0.48 | -2.0 | 0.76 | 1.8 | 2.49 | -5.41 | **0.000** |
| *Columba livia* | 908 | -56.3 | 0.1 | 0.0 | 1.0 | 1.8 | 11.2 | 3.80 | -2.06 | 6.2 | 0.85 | 2.7 | 2.47 | 2.31 | 0.021 |
| *Columba oenas* | 205 | -184.7 | 0.0 | 0.0 | 4.5 | 5.4 | 1.8 | -1.11 | 0.28 | -2.0 | 0.38 | 1.5 | 1.84 | -7.97 | **0.000** |
| *Columba palumbus* | 1377 | -366.1 | 2.9 | 5.6 | 8.1 | 10.4 | 24.7 | 13.32 | -13.34 | 3.3 | 0.67 | 2.4 | 1.96 | -2.35 | 0.019 |
| *Corvus corax* | 1151 | -4.1 | 3.0 | 2.4 | 0.8 | 0.2 |  |  |  |  | 0.89 | 2.1 | 2.47 | -5.97 | **0.000** |
| *Corvus corone* | 1090 | -41.7 | 16.9 | 7.9 | 2.1 | 1.4 | 31.8 | 3.12 | -7.30 | 1.4 | 0.77 | 1.6 | 2.06 | -16.02 | **0.000** |
| *Corvus monedula* | 466 | -6.0 | 0.0 | 0.0 | 0.1 | 0.3 |  |  |  |  | 0.91 | 2.8 | 2.48 | 2.16 | 0.031 |
| *Cyanistes caeruleus* | 1426 | -163.8 | 2.3 | 2.3 | 2.8 | 4.9 | 32.4 | 9.53 | -8.01 | 4.0 | 0.84 | 2.5 | 2.20 | -1.76 | 0.078 |
| *Cyanopica cooki* | 410 | -340.3 | 0.0 | 0.2 | 8.1 | 9.7 | 13.6 | 9.02 | -7.73 | 3.9 | 0.68 | 3.2 | 1.99 | 6.21 | **0.000** |
| *Dendrocopos major* | 1026 | -47.8 | 7.3 | 5.3 | 2.4 | 1.5 | 11.7 | -1.92 | 1.41 | -2.0 | 0.82 | 1.8 | 2.37 | -10.28 | **0.000** |
| *Dendrocopos minor* | 109 | -123.5 | 0.0 | 0.0 | 0.0 | 3.7 | 0.8 | 0.59 | -0.54 | 3.7 | 0.64 | 3.0 | 1.80 | 2.41 | 0.018 |
| *Dryocopus martius* | 56 | -209.6 | 0.0 | 0.0 | 0.0 | 6.1 | 0.3 | -0.62 | 0.40 | -2.0 | 0.26 | 0.5 | 1.67 | -9.41 | **0.000** |
| *Elanus caeruleus* | 126 | -78.0 | 0.0 | 0.0 | 1.1 | 2.4 | 1.0 | 0.57 | -0.08 | 10.0 | 0.56 | 3.8 | 2.24 | 6.17 | **0.000** |
| *Emberiza calandra* | 1096 | -211.4 | 0.7 | 0.9 | 3.8 | 6.2 | 26.0 | 10.05 | -7.34 | 4.6 | 0.82 | 2.7 | 2.32 | 1.58 | 0.115 |
| *Emberiza cia* | 1105 | -36.0 | 4.5 | 4.9 | 3.2 | 1.2 | 15.3 | -3.61 | 2.74 | -2.0 | 0.77 | 1.9 | 2.43 | -8.97 | **0.000** |
| *Emberiza cirlus* | 934 | -67.0 | 1.8 | 2.0 | 2.3 | 2.1 | 12.2 | 1.50 | -3.30 | 1.5 | 0.74 | 2.0 | 2.27 | -7.02 | **0.000** |
| *Emberiza citrinella* | 317 | -280.8 | 0.0 | 2.4 | 8.2 | 8.1 | 2.8 | -2.17 | 0.70 | -2.0 | 0.34 | 1.0 | 1.93 | -14.16 | **0.000** |
| *Emberiza schoeniclus* | 305 | 4.0 | 0.0 | 0.0 | 0.0 | 0.0 |  |  |  |  | 1.00 | 2.7 | 2.56 | 1.11 | 0.268 |
| *Erithacus rubecula* | 1604 | -652.7 | 13.7 | 19.2 | 18.5 | 17.7 | 43.3 | 18.94 | -11.34 | 5.6 | 0.82 | 3.4 | 2.28 | 15.40 | **0.000** |
| *Falco columbarius* | 303 | -146.4 | 0.0 | 0.0 | 0.9 | 4.4 | 1.7 | 0.00 | -0.37 | 0.0 | 0.69 | 1.7 | 1.96 | -7.19 | **0.000** |
| *Falco peregrinus* | 283 | 4.0 | 0.0 | 0.0 | 0.0 | 0.0 |  |  |  |  | 1.00 | 2.5 | 2.42 | -0.62 | 0.539 |
| *Falco tinnunculus* | 1310 | -35.9 | 2.3 | 3.3 | 2.8 | 1.2 | 11.8 | -2.27 | 2.42 | 10.0 | 0.72 | 2.8 | 2.76 | 2.61 | 0.009 |
| *Fringilla coelebs* | 1649 | -88.6 | 1.4 | 2.2 | 2.1 | 2.7 | 49.6 | 6.46 | -5.68 | 3.8 | 0.92 | 2.6 | 2.37 | 1.29 | 0.197 |
| *Fringilla montifringilla* | 332 | -104.8 | 0.0 | 0.5 | 2.7 | 3.2 | 2.3 | -1.06 | 0.74 | -2.0 | 0.61 | 1.5 | 2.33 | -8.15 | **0.000** |
| *Galerida cristata* | 1131 | -257.3 | 0.3 | 2.1 | 4.1 | 7.5 | 34.6 | 11.32 | -5.41 | 7.0 | 0.84 | 3.0 | 2.51 | 5.38 | **0.000** |
| *Galerida theklae* | 720 | -291.0 | 0.0 | 2.6 | 5.2 | 8.4 | 20.0 | 8.88 | -3.43 | 8.7 | 0.76 | 3.4 | 2.43 | 9.34 | **0.000** |
| *Garrulus glandarius* | 1098 | -101.6 | 1.5 | 2.0 | 3.3 | 3.1 | 19.0 | 4.45 | -5.96 | 2.5 | 0.76 | 2.3 | 2.19 | -3.48 | **0.001** |
| *Grus grus* | 193 | -109.2 | 0.0 | 0.0 | 1.2 | 3.3 | 2.8 | 2.08 | -1.76 | 4.0 | 0.65 | 3.4 | 1.77 | 6.68 | **0.000** |
| *Gyps fulvus* | 800 | -39.1 | 3.1 | 3.8 | 2.4 | 1.3 | 10.8 | -0.13 | -1.48 | -0.3 | 0.77 | 1.9 | 2.21 | -8.58 | **0.000** |
| *Hieraaetus fasciatus* | 96 | -518.3 | 0.0 | 0.0 | 1.9 | 14.3 | 0.4 | 0.15 | 0.30 | 10.0 | 0.32 | 4.4 | 2.31 | 7.73 | **0.000** |
| *Lanius meridionalis* | 1048 | -372.1 | 0.0 | 2.1 | 7.1 | 10.5 | 11.0 | 4.39 | -1.40 | 9.9 | 0.73 | 3.2 | 2.43 | 8.67 | **0.000** |
| *Lophophanes cristatus* | 905 | -264.4 | 0.2 | 1.3 | 5.1 | 7.6 | 19.5 | -9.38 | 6.24 | -2.0 | 0.59 | 1.9 | 2.55 | -7.48 | **0.000** |
| *Loxia curvirostra* | 393 | -222.6 | 0.0 | 3.4 | 5.3 | 6.5 | 7.6 | -6.64 | 6.37 | 10.0 | 0.45 | 1.7 | 2.82 | -5.98 | **0.000** |
| *Lullula arborea* | 881 | -236.2 | 0.9 | 1.4 | 3.8 | 6.9 | 18.1 | 11.37 | -8.91 | 4.3 | 0.72 | 2.7 | 2.15 | 2.25 | 0.025 |
| *Melanocorypha calandra* | 535 | -70.1 | 0.0 | 2.3 | 2.5 | 2.2 | 18.7 | -4.50 | 0.70 | -2.0 | 0.63 | 1.9 | 2.18 | -7.06 | **0.000** |
| *Milvus milvus* | 794 | -167.2 | 2.2 | 5.1 | 4.4 | 4.9 | 14.9 | -1.12 | -2.56 | -1.5 | 0.65 | 1.7 | 2.04 | -12.20 | **0.000** |
| *Monticola solitarius* | 223 | -671.3 | 0.0 | 0.7 | 14.3 | 18.1 | 1.4 | 0.70 | 0.31 | 10.0 | 0.41 | 4.8 | 2.06 | 16.02 | **0.000** |
| *Motacilla alba* | 1495 | -1381.8 | 21.5 | 28.6 | 32.5 | 33.7 | 23.9 | 10.40 | 1.13 | 10.0 | 0.51 | 4.1 | 2.41 | 25.44 | **0.000** |
| *Motacilla cinerea* | 690 | -138.4 | 0.0 | 0.6 | 2.6 | 4.1 | 4.0 | 1.52 | -0.78 | 6.5 | 0.83 | 3.2 | 2.36 | 6.97 | **0.000** |
| *Oenanthe leucura* | 145 | -698.5 | 0.0 | 0.0 | 11.7 | 18.8 | 1.5 | 0.49 | 1.06 | 10.0 | 0.32 | 4.8 | 2.26 | 11.90 | **0.000** |
| *Otis tarda* | 143 | -192.8 | 0.0 | 0.0 | 0.2 | 5.7 | 2.5 | -2.33 | 0.87 | -2.0 | 0.30 | 1.3 | 1.47 | -10.20 | **0.000** |
| *Parus major* | 1614 | -138.0 | 2.4 | 3.3 | 4.1 | 4.1 | 38.3 | 7.63 | -5.08 | 5.0 | 0.91 | 2.8 | 2.34 | 3.92 | **0.000** |
| *Passer domesticus* | 1553 | -732.7 | 14.7 | 18.2 | 18.3 | 19.6 | 25.1 | 8.63 | -0.23 | 10.0 | 0.60 | 3.7 | 2.49 | 18.64 | **0.000** |
| *Passer hispaniolensis* | 231 | -266.1 | 0.0 | 0.0 | 4.0 | 7.7 | 4.8 | 3.30 | -1.26 | 8.7 | 0.68 | 3.9 | 1.54 | 13.60 | **0.000** |
| *Passer montanus* | 667 | -53.8 | 0.0 | 3.2 | 0.6 | 1.7 | 7.0 | -2.39 | 1.44 | -2.0 | 0.65 | 2.0 | 2.37 | -5.59 | **0.000** |
| *Periparus ater* | 934 | -72.9 | 1.4 | 1.4 | 2.2 | 2.2 | 25.4 | -7.96 | 7.54 | 10.0 | 0.67 | 2.1 | 2.54 | -5.64 | **0.000** |
| *Petronia petronia* | 586 | -358.2 | 0.0 | 7.0 | 9.4 | 10.2 | 6.9 | -7.38 | 4.54 | -2.0 | 0.38 | 1.2 | 2.17 | -14.63 | **0.000** |
| *Phoenicurus ochruros* | 1389 | -1401.6 | 15.4 | 23.0 | 30.2 | 34.0 | 18.6 | 10.64 | -1.10 | 10.0 | 0.53 | 4.1 | 2.30 | 24.58 | **0.000** |
| *Phylloscopus collybita* | 1316 | -1594.4 | 25.4 | 33.4 | 37.2 | 37.7 | 21.2 | 9.25 | 3.95 | 10.0 | 0.43 | 4.5 | 2.34 | 30.50 | **0.000** |
| *Pica pica* | 1353 | -268.8 | 2.9 | 4.9 | 7.9 | 7.8 | 31.7 | 18.39 | -18.12 | 3.4 | 0.66 | 2.5 | 2.00 | -1.09 | 0.278 |
| *Picus viridis* | 1201 | -29.1 | 1.6 | 0.7 | 0.4 | 1.0 | 12.2 | 0.09 | -1.27 | 0.2 | 0.85 | 2.3 | 2.32 | -4.56 | **0.000** |
| *Poecile palustris* | 141 | -75.5 | 0.0 | 0.0 | 0.8 | 2.3 | 2.0 | 0.17 | -0.55 | 1.0 | 0.70 | 1.8 | 1.61 | -5.27 | **0.000** |
| *Prunella collaris* | 70 | -92.5 | 0.0 | 0.0 | 0.0 | 2.8 | 0.3 | -0.59 | 0.45 | -2.0 | 0.29 | 2.0 | 2.74 | -1.63 | 0.107 |
| *Prunella modularis* | 865 | -234.3 | 0.8 | 1.6 | 5.1 | 6.8 | 16.1 | 9.65 | -8.81 | 3.7 | 0.68 | 2.8 | 2.06 | 3.22 | 0.001 |
| *Pterocles alchata* | 86 | -105.8 | 0.0 | 0.0 | 0.0 | 3.2 | 0.8 | 0.61 | -0.56 | 3.7 | 0.62 | 2.5 | 1.37 | -0.20 | 0.840 |
| *Pterocles orientalis* | 150 | -94.4 | 0.0 | 0.0 | 0.0 | 2.9 | 1.7 | 0.98 | -0.97 | 3.4 | 0.66 | 2.6 | 2.00 | 0.02 | 0.987 |
| *Ptyonoprogne rupestris* | 366 | -1643.3 | 0.0 | 18.0 | 33.0 | 38.6 | 3.8 | 0.59 | 3.25 | 10.0 | 0.32 | 5.5 | 2.34 | 24.20 | **0.000** |
| *Pyrrhocorax pyrrhocorax* | 463 | -105.2 | 0.0 | 1.8 | 2.1 | 3.2 | 4.6 | 0.91 | -1.56 | 2.0 | 0.71 | 2.0 | 2.07 | -6.05 | **0.000** |
| *Pyrrhula pyrrhula* | 402 | -115.0 | 0.0 | 1.0 | 3.5 | 3.5 | 5.0 | -0.20 | -0.98 | -0.7 | 0.67 | 1.9 | 2.03 | -6.90 | **0.000** |
| *Regulus ignicapilla* | 1123 | -212.1 | 1.0 | 1.6 | 3.9 | 6.2 | 16.9 | 7.01 | -5.16 | 4.5 | 0.81 | 2.6 | 2.21 | 0.18 | 0.858 |
| *Regulus regulus* | 492 | -623.4 | 0.0 | 9.0 | 12.2 | 17.0 | 5.1 | -4.51 | 2.23 | -2.0 | 0.37 | 0.8 | 2.16 | -17.68 | **0.000** |
| *Remiz pendulinus* | 63 | -227.6 | 0.0 | 0.0 | 0.0 | 6.6 | 0.2 | -0.05 | 0.35 | 10.0 | 0.27 | 4.2 | 2.56 | 5.15 | **0.000** |
| *Saxicola rubicola* | 1365 | -1238.9 | 19.7 | 26.2 | 29.4 | 30.8 | 18.8 | 10.99 | -3.18 | 10.0 | 0.64 | 4.2 | 2.39 | 25.39 | **0.000** |
| *Serinus citrinella* | 114 | -654.7 | 0.0 | 0.0 | 7.2 | 17.7 | 1.3 | -2.65 | 1.71 | -2.0 | 0.26 | -0.2 | 1.53 | -19.29 | **0.000** |
| *Serinus serinus* | 1224 | -1660.7 | 15.2 | 29.5 | 37.2 | 38.9 | 26.4 | 14.43 | 1.75 | 10.0 | 0.46 | 4.5 | 2.19 | 31.39 | **0.000** |
| *Sitta europaea* | 640 | 3.4 | 0.0 | 2.0 | 0.2 | 0.0 |  |  |  |  | 0.84 | 2.4 | 2.45 | -1.86 | 0.063 |
| *Streptopelia decaocto* | 911 | -919.8 | 4.8 | 12.0 | 20.2 | 23.9 | 9.8 | 4.96 | 0.75 | 10.0 | 0.47 | 4.2 | 2.29 | 21.45 | **0.000** |
| *Sturnus unicolor* | 1445 | -335.0 | 2.0 | 4.9 | 7.4 | 9.5 | 27.5 | 14.27 | -10.18 | 4.7 | 0.78 | 3.1 | 2.37 | 8.06 | **0.000** |
| *Sturnus vulgaris* | 629 | -455.2 | 0.0 | 3.7 | 8.4 | 12.7 | 10.2 | 5.37 | -0.10 | 10.0 | 0.51 | 3.8 | 2.28 | 14.11 | **0.000** |
| *Sylvia atricapilla* | 1000 | -980.2 | 13.9 | 21.0 | 24.3 | 25.3 | 20.8 | 11.82 | 0.45 | 10.0 | 0.48 | 4.5 | 2.00 | 29.94 | **0.000** |
| *Sylvia melanocephala* | 922 | -1498.4 | 20.1 | 34.9 | 37.8 | 35.9 | 27.0 | 15.27 | 0.77 | 10.0 | 0.48 | 4.8 | 2.14 | 31.88 | **0.000** |
| *Sylvia undata* | 1096 | -185.9 | 0.7 | 2.7 | 5.0 | 5.5 | 19.6 | 2.95 | 0.93 | 10.0 | 0.67 | 3.2 | 2.54 | 8.34 | **0.000** |
| *Tetrax tetrax* | 120 | -103.9 | 0.0 | 0.0 | 0.0 | 3.1 | 0.9 | 0.62 | -0.57 | 3.6 | 0.63 | 2.4 | 1.61 | -0.74 | 0.462 |
| *Troglodytes troglodytes* | 1052 | -382.1 | 0.8 | 0.7 | 5.6 | 10.8 | 31.9 | 14.02 | -7.31 | 6.4 | 0.81 | 2.9 | 2.27 | 4.87 | **0.000** |
| *Turdus iliacus* | 712 | -26.2 | 0.0 | 0.1 | 0.0 | 0.9 | 8.5 | 0.10 | -1.02 | 0.3 | 0.83 | 2.3 | 2.34 | -2.76 | **0.006** |
| *Turdus merula* | 1634 | -187.5 | 3.7 | 3.7 | 4.5 | 5.5 | 41.0 | 7.52 | -4.63 | 5.4 | 0.91 | 2.9 | 2.45 | 5.88 | **0.000** |
| *Turdus philomelos* | 1488 | -350.0 | 6.5 | 7.2 | 8.8 | 10.0 | 32.2 | 16.13 | -10.89 | 4.9 | 0.79 | 3.2 | 2.21 | 11.30 | **0.000** |
| *Turdus pilaris* | 372 | -653.2 | 0.0 | 9.9 | 16.0 | 17.7 | 2.9 | -3.18 | 1.33 | -2.0 | 0.28 | 0.7 | 1.84 | -19.97 | **0.000** |
| *Turdus torquatus* | 58 | -66.6 | 0.0 | 0.0 | 0.0 | 2.1 | 0.3 | -0.73 | 0.57 | -2.0 | 0.31 | 2.0 | 2.70 | -1.66 | 0.102 |
| *Turdus viscivorus* | 1206 | -330.8 | 4.1 | 4.2 | 6.7 | 9.4 | 20.5 | -5.07 | 0.04 | -2.0 | 0.59 | 1.7 | 2.27 | -12.75 | **0.000** |
| *Upupa epops* | 602 | -981.5 | 3.1 | 17.9 | 23.3 | 25.3 | 9.7 | 5.27 | 1.39 | 10.0 | 0.43 | 4.5 | 1.71 | 28.52 | **0.000** |
